# Outbreak of *Listeria monocytogenes* in an urban poultry flock

**DOI:** 10.1186/1746-6148-9-204

**Published:** 2013-10-11

**Authors:** Rocio Crespo, Michael M Garner, Sharon G Hopkins, Devendra H Shah

**Affiliations:** 1 Avian Health and Food Safety Laboratory, Washington Animal Disease Diagnostic Laboratory, Washington State University, 2607 West Pioneer, Puyallup, WA 98371, USA; 2 Northwest ZooPath, 654 W. Main St., Monroe, WA 98272, USA; 3 Public Health–Seattle & King County, 401 5th Avenue, Ste 1100, Seattle, WA 98104, USA; 4 Department of Veterinary Microbiology, Washington State University, Pullman, WA 99164-7040, USA; 5 Pathology and Paul Allen School for Global Animal Health, Washington State University, Pullman, WA 99164-7040, USA

## Abstract

**Background:**

*Listeria monocytogenes* infection is most commonly recognized in ruminants, including cattle, sheep, and goats; but it is rarely diagnosed in poultry. This report describes an outbreak of *L. monocytogenes* in a backyard poultry flock. Also, it points out the importance of collaboration between veterinarians and public health departments and the possible implications of zoonotic diseases.

**Case presentation:**

Depression, lack of appetite, labored breathing, and increased mortality were noted for 5 months in several affected birds within the flock. The pathologic changes in the internal organs of infected birds included severe myocarditis, pericarditis, pneumonia, hepatitis, and splenitis. No lesions were noted in the brain. Gram-positive organisms were seen in histologic sections of the heart and spleen. *Listeria monocytogenes* was detected by real time PCR from formalin fixed heart and spleen, and was isolated from fresh lung, spleen, and liver. This isolate was identified as *L. monocytogenes* serotype 4b by 16S rDNA sequencing and by PCR-based serotyping assay.

**Conclusions:**

This is the first report describing outbreak of *L. monocytogenes* in backyard poultry flock in Washington State and use of molecular methods to confirm *L. monocytogenes* infection from formalin fixed tissues.

## Background

Listeriosis is caused by the bacterium *Listeria monocytogenes* and has a worldwide distribution. Although many species of birds are susceptible to infection, clinical disease in birds is rare. Young birds are most susceptible. *L. monocytogenes* infections in chickens occur in two forms: encephalitic or septicemic form. The encephalitic form is characterized by neurologic signs such as depression, incoordination and torticollis; whereas the septicemic form is characterized by diarrhea and emaciation.

*L. monocytogenes* has been isolated from poultry, poultry products, and ready-to-eat poultry meat [1-3]. One study showed that *L. monocytogenes* was isolated from >40% of the organic as well as non-organic commercial chicken meat in Maryland [4]. The vast majority of serotypes included 1/2a, 1/2b and 4b. Interestingly, out of more than 14 reported serotypes of *L. monocytogenes,* these three serotypes cause most of the clinical cases [5]. Several studies have reported that handling and consumption of contaminated raw broiler meat is an important risk factor for human infection. Because of the epidemiological importance of certain serotypes of *L. monocytogenes* to human health and potential transmission of the pathogen from poultry to humans, accurate detection of *L. monocytogenes* followed by subtyping methods to identify the specific serotype or genotype involved in outbreaks is essential. This report describes an unusual presentation of listeriosis in adult chickens and the systematic use of molecular tests performed to diagnose listeriosis in this backyard poultry flock. Also, it stresses the importance of being aware of potentially zoonotic diseases.

## Case presentation

### Clinical history

Several chickens from a backyard flock (Seattle, WA) were presented to a private veterinarian. The affected flock consisted of 20 chickens that were approximately 8 months of age at the time of presentation. During the course of the previous five months, a total of 7 birds had died and other 5 had shown clinical signs of illness. Most birds stopped producing eggs. The clinical signs included depression, anorexia and panting. The owner medicated the flock with sulfadimethoxine sodium in the water (1 fl oz of 12.5% Sulmet per gallon of drinking water; Fort Dodge Animal Health, Fort Dodge, IA) for 5 days. Despite this treatment, no improvement in clinical signs or egg production was noticed. The owner provided commercial layer feed (Payback®, CHS Inc., Sioux Falls, SD) purchased from the local feed store in Issaquah, WA. The owner also reported occasionally feeding some fresh garden produce grown on the premises, but no processed foods (e.g., cold cuts, hot dogs, cheese) were offered to birds.

### Pathology

The owner submitted a dead chicken (the 6^th^ one that died) to a local veterinarian for necropsy. The veterinarian collected several tissues (including heart, lung, air sac, intestine, pancreas, and liver) and submitted them for histologic examination to a private veterinary diagnostic laboratory. Two weeks later, another bird, an 8-month-old female chicken, died (the 7^th^ death in the flock) and was submitted to the Avian Health and Food Safety Laboratory (AHFSL) for necropsy. The bird submitted to AHFSL had small comb fecal pasting around the cloaca. The bird was in good body condition and weighed 2,065 grams. The gross findings were 1-2 cc serous fibrinous pericardial effusion, thickening and opacity of pericardium with multiple pale foci throughout the myocardium. Pulmonary congestion, fibrinous exudate on the pleura, and thickening of air sacs were noted. The liver and spleen were enlarged and mottled.

Portions of the heart, liver, lung, kidney, gizzard, proventriculus, intestine, pancreas, ovary, oviduct, thyroid, parathyroid skeletal muscle, peripheral nerve, and brain were collected for histopathology. Tissues were fixed in 10% buffered neutral formalin, routinely processed, embedded in paraffin, sectioned at 4 μm, stained with hematoxylin and eosin, and examined by light microscopy. Additionally, sections of heart, liver and spleen were stained with modified Brown-Hopps tissue Gram stain [6].

**Figure 1:**
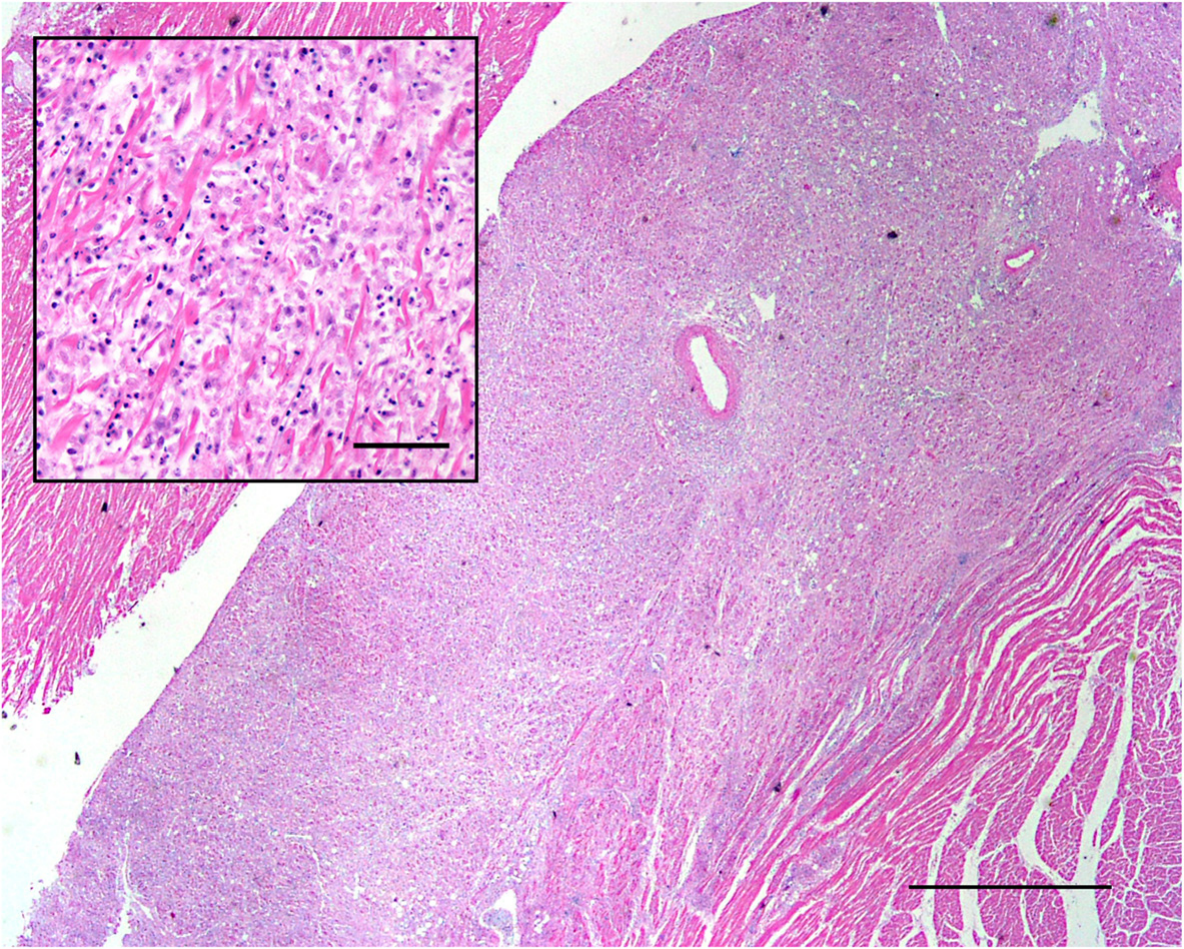
**Extensive chronic-active myocardial inflammation.** Histologic section of the heart showing extensive inflammation. H&E. Bar = 1 mm. Insert: higher magnification of the heart showing the inflammatory cell infiltration and disruption of myofibers. Inflammatory cells consisted mostly of lymphocytes and macrophages with scattered heterophils. H&E. Bar = 50 μm.

**Figure 2:**
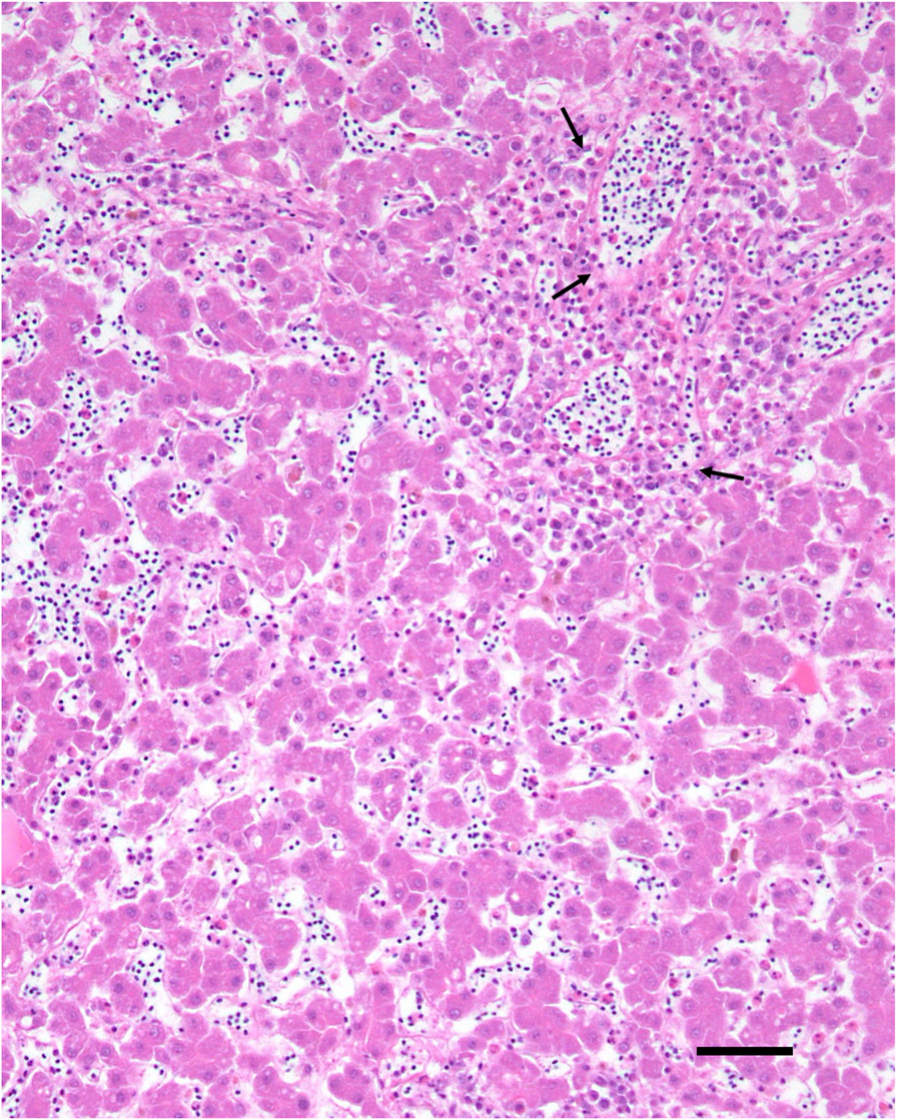
**Periportal hepatic inflammation.** Histologic section of the liver showing accumulation of heterophils and macrophages mostly around portal areas (arrows). H&E. Bar = 50 μm.

**Figure 3:**
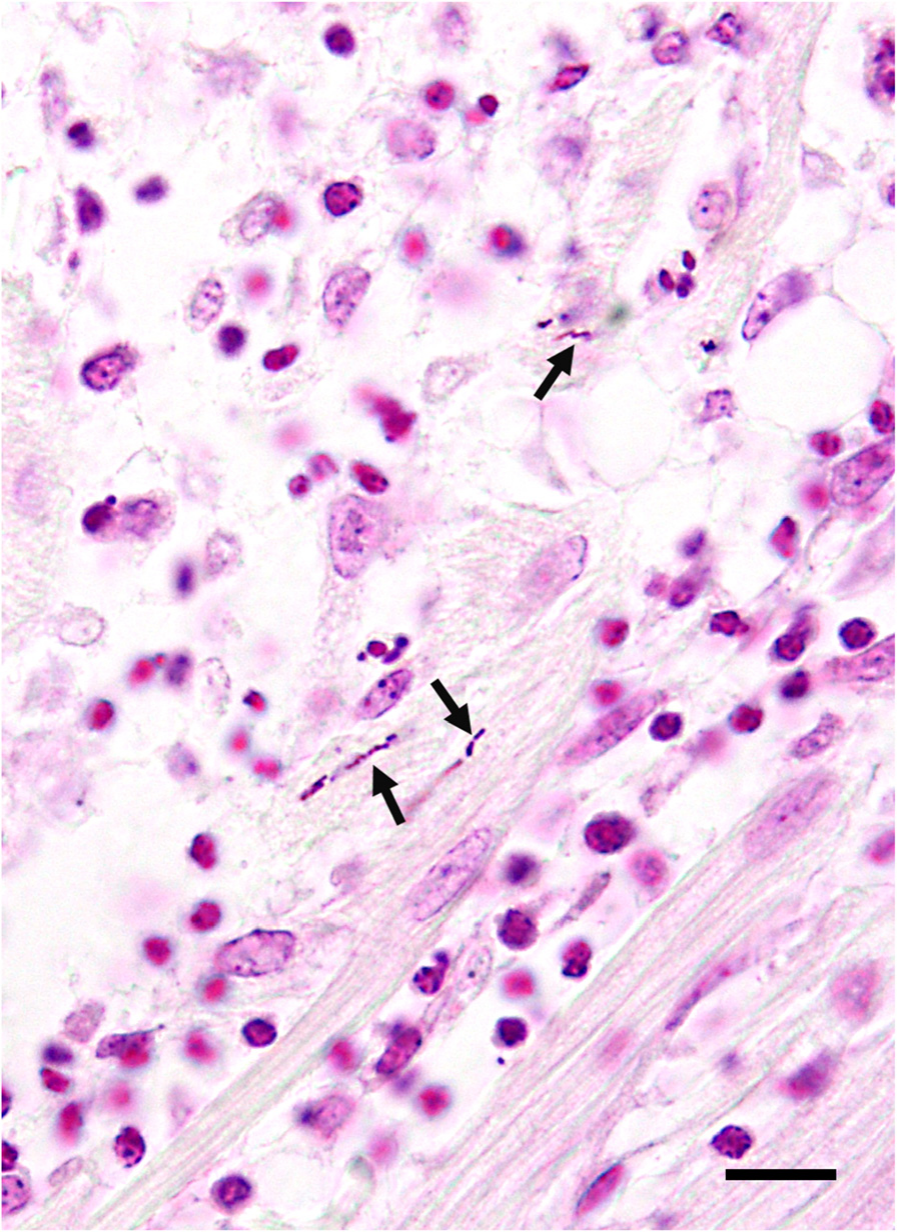
**Gram positive bacteria in the heart.** Histologic section of the heart showing inflammation and presence of Gram-positive rod-shaped bacteria (arrows) between the myofibers. Modified Brown-Hopps gram stain. Bar = 10 μm.

Histologic findings were similar in the two birds submitted separately. The most prominent lesion was extensive inflammation of the heart (Figure 1), consisted of lymphocytes, macrophages, numerous multinucleated giant cells, and occasional heterophils. The lumen of larger blood vessels contained numerous large lymphocytes and scattered heterophils. In the liver there were random, small necrotic foci and accumulation of heterophils and macrophages (Figure 2). Lung sections revealed interstitial pneumonia, with infiltration of heterophils in the air capillaries and edema accumulating in the interparabronchial spaces. Lesions in the pancreas consisted of mild lymphocytic inflammation around the ducts. Spleen sections revealed severe lymphoid depletion, random accumulation of fibrin in the white pulp, and several macrophages contained intracytoplasmic cellular debris. Rod-shaped, Gram-positive bacteria were noted in the heart (Figure 3) and spleen.

### Bacteriology

Heart, liver, spleen and lung specimens were inoculated on blood and McConkey’s agar (Remel, Lenaxa, KS) plates and incubated aerobically at 37°C for a minimum of up to 48 h. Cultures were examined for bacterial growth at approximately 24 and 48 h post-inoculation. Additionally, multiple sections of intestine were collected and cultured for *Salmonella spp*. The intestinal contents were selectively enriched in tetrathionate (TT) Hajna (BD Worlwide, Franklin Lakes, NJ) broth at 40°C for 20-24 hr. After enrichment, TT broth was plated on brilliant green with novobiocin and xylose-lysine-tergitol agar 4 agars (Remel) and the plates were incubated at 35°C for an additional 24 h.

Identical small round colonies with a narrow zone of β-hemolysis on the blood agar were isolated in pure form from liver, spleen, and lung. The bacteria were identified as Gram-positive rods, catalase-positive and oxidase-negative. The liver isolate was biochemically characterized as *L. monocytogenes* by testing with the API Coryne kit (bioMérieux, Inc., Hazelwood, MO) and Christie–Atkins–Munch–Peterson (CAMP) test.

### Molecular characterization

The isolate was confirmed to be *L. monocytogenes* by the real-time polymerase chain reaction (RT-PCR) using iQ-Check Listeria monocytogenes II kit (BioRad, Hercules, CA) according to the manufacturer’s instructions. In addition, the genomic DNA was extracted from paraffin sections of heart from both birds, and the liver and the spleen from the bird submitted at AHFSL using a QIAamp® DNA Mini Kit (Qiagen, Valencia, CA) and tested by RT-PCR. *L. monocytogenes* was detected from both fixed sections of heart (Ct = 28.35 and 38.45) and the single fixed section of spleen (Ct = 32.48). No bacteria were isolated from the heart. None of the samples were positive for *Salmonella* sp.

**Figure 4:**
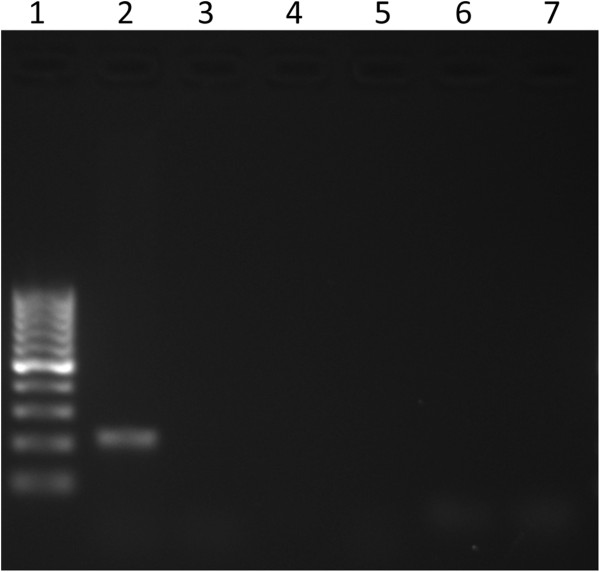
**Molecular serotyping of L. monocytogenes.** Results of PCR-based serotyping of *L. monocytogenes*. Lane 1: 100-bp ladder; lane 2: D1 primers; lane 3: D2 primers; lane 4: FlaA primers; lane 5: GLT primers; lane 6: MAMA-C primers; lane 7: no template control. Positive amplification with D1 primer pair only. This suggests that the *L. monocytogenes* isolate is serotype 4b [8].

A complete ORF of 16S rDNA (~1500 bp) was PCR amplified, cloned in pGEM-T Easy vector system (Promega, Madison, WI) and sequenced using protocols described previously [7]. The sequences were edited, aligned and phyologenetic analysis (data not shown) was performed as described previously [7]. The 16S rRNA gene sequence from *L. monocytogenes* strain showed 99.9% similarity with several *L. monocytogenes* strains in the GenBank database, but serotype of this isolate could not be determined based on the 16S rDNA sequence similarity. Consequently, PCR based serotyping was performed following the procedures described previously using following sets of primers that amplify variable regions of *L. monocytogenes* genome: D1, D2, FlaA, GLT and MAMA-C [8]. These primer pairs can accurately classify *L. monocytogenes* into specific serotype. The *L. monocytogenes* isolate in this study tested PCR positive only by one primer set (D1), but negative for all the other primer pairs tested (Figure 4). These results suggested that the isolate belonged to *L. monocytogenes* serotype 4b.

## Discussion

In animals and humans the most common *Listeria* infections are caused by three serotypes: 1/2a, 1/2b, and 4b. PCR-based serotyping of the *L. monocytogenes* isolate in the current study identified this as serotype 4b, one of the most pathogenic serovars that is known to cause majority of human infections [9]. Experimental studies have shown that serotype 4b strains may have increased virulence compared with other serotypes [9] and are often responsible for invasive disease outbreaks in mammals. This serotype is also found in poultry meat [4]. In addition, serotype 4b has been isolated from a chicken flock with encephalitis [10]. However, no signs of central nervous system disease were observed in the current outbreak.

In the current case, *L. monocytogens* were isolated from the lung, liver and spleen, but not from the heart. On the other hand the bacteria were detected by RT-PCR from fixed heart and spleen. Molecular techniques have been used to detect *L. monocytogenes* in enriched samples or pure cultures. The limit of detection for the commercial real time PCR kit used in this study is 1,000 coliform forming units. The Ct value for qPCR for this bird was 38.45 which indicated that this bird likely had low numbers of *L. monocytogenes* in heart and that could have led to failure of isolation. It is possible that cold-enrichment might have helped recovering the bacteria from the heart. However cold-enrichment was not conducted because all other samples were positive.

The exact source of infection for this flock is unknown. Possible sources of the *Listeria* infection include feces, soil, decaying plant material, feed, and water. Neither the environment where the chickens were housed nor the commercial ration or drinking water were collected or tested. Furthermore, various species of mammals and birds may be infected with *Listeria* and may serve as asymptomatic carriers [11].

The first clinical signs in the flock appeared when investigation from a multistate outbreak of listeriosis linked to whole cantaloupes was ongoing [12]. The number of outbreak-associated deaths in the mentioned outbreak was 33 people. According to the history and confirmed by the King’s County Public Health investigation, the chicken flock was fed a commercial layer’s ration and occasional fresh produce grown in the owner’s garden. The birds did not have access to compost or kitchen leftovers that may be contaminated with listeria (e.g. like cold cuts, hot dogs, soft cheeses, or cantaloupes). Conjunctivitis due to *L. monocytogenes* has been reported in individuals handling apparently healthy but infected chickens [13]. Human infections have also resulted from the consumption of contaminated poultry or ready-to-eat poultry products. Although the family continued to consume the eggs produced by the flock during the outbreak, no family member was ever sick during the outbreak or diagnosed with Listeriosis.

The sick birds were treated intramuscularly with 25 mg/kg of enrofloxacin (Bayer Corp., Pittsburgh, PA) for 5 days. All the birds recovered. Enrofloxacin is a fluoroquinolone antibiotic used for the treatment of a wide variety of bacterial infections in companion animals and horses. In 2005, the Food and Drug Adminstraton withdrew approval of enrofloxacin for use in water to treat flocks of poultry, as this practice was noted to promote the evolution of fluoroquinolone-resistant strains of the bacterium Campylobacter, a human pathogen [14]. Unlike commercial poultry, these birds or their eggs were not sold for food. The veterinarian educated the clients regarding the potential of contamination of eggs with the antibiotic and recommended not to consume the eggs laid by treated hens.

## Conclusion

This is the first report of outbreak of listeriosis in the backyard poultry flock in Washington State. In addition, this paper describes the use of real time PCR, for the first time, to confirm *L. monocytogenes* infection from formalin fixed tissues. Because most of human infections are caused by three major serotypes (serotyping must be performed to identify the specific serotypes of *Listeria* (1/2a, 1/2b and 4b), serotyping must be undertaken to determine potential public health risk associated with exposure to infected birds. It is important to note that most reported outbreaks of listeriosis in poultry involve relatively very young birds. Systemic listeriosis in birds older than 5 months of age described in this report is relatively rare, but the current outbreak suggests that veterinarians and public health officials must be vigilant and should raise suspicion for listeriosis irrespective of age of the chickens. Finally, because antibiotics may remain in meat and other animal products, it is essential to educate the owners of urban poultry on these potential risks.

## Abbreviations

AHFSL: Avian health and food safety laboratory; TT: Tetrathionate hajna broth.

## Competing interests

The authors declare that they have no competing interests.

## Authors’ contributions

RC performed the necropsy, the histologic examination, isolation, and PCR from fixed tissues, reviewed the literature and prepared the manuscript. MG performed the histologic examination and prepared the photos. SH performed the field investigation and interviewed the owner. DS performed the molecular characterization and serotype-specific identification of *Listeria* isolate. All authors read and approved the final manuscript.
